# Immunotherapy-based adjuvant treatment after neoadjuvant immunochemotherapy in esophageal squamous cell carcinoma according to pathological response

**DOI:** 10.3389/fimmu.2026.1802037

**Published:** 2026-06-03

**Authors:** Chenyu Wang, Xiaoran Geng, Xinyu Cheng, Runchuan Ren, Linzhi Jin, Zhaojie Sheng, Shenglei Li, Meiling Zang, Yaowen Zhang

**Affiliations:** 1 Department of Radiation Oncology, Anyang Tumor Hospital, The Affiliated Anyang Tumor Hospital of Henan University of Science and Technology, Henan Medical Key Laboratory of Precise Prevention and Treatment of Esophageal Cancer, Anyang, China; 2 Research and Teaching Department, Anyang Tumor Hospital, Anyang, China

**Keywords:** esophageal squamous cell carcinoma, immunotherapy-based adjuvant treatment, neoadjuvant immunochemotherapy, pathological complete response, pathological downstaging

## Abstract

**Background:**

Neoadjuvant immunochemotherapy (NICT) is increasingly used in patients with locally advanced resectable esophageal squamous cell carcinoma (ESCC). However, optimal postoperative adjuvant treatment strategies remain unclear. This study aimed to evaluate the efficacy of immunotherapy-based adjuvant treatment in patients with ESCC after NICT according to pathological response status.

**Methods:**

We retrospectively analyzed 260 patients with locally advanced ESCC who underwent radical resection after NICT between 2019 and 2023. Patients were first stratified by pathological response status [pathological complete response (pCR) vs. non-pCR], and immunotherapy-based adjuvant treatment was subsequently compared with observation within each stratum. Baseline imbalances were adjusted using propensity score matching (PSM) and inverse probability of treatment weighting (IPTW). Disease-free survival (DFS) and overall survival (OS) were assessed using the Kaplan–Meier method, log-rank test, and Cox proportional hazards models.

**Results:**

Of the 260 patients, 84 (32.3%) achieved pCR, and 81 (31.2%) received immunotherapy-based adjuvant treatment. The median follow-up was 36.8 months. In the pCR cohort, immunotherapy-based adjuvant treatment did not significantly improve 3-year DFS or OS compared with observation (80.8% vs. 90.1%, P = 0.065; 96.2% vs. 91.8%, P = 0.486), with consistent results in the PSM and IPTW analyses. In the overall non-pCR cohort, immunotherapy-based adjuvant treatment likewise did not confer significant survival benefits (3-year DFS: 71.9% vs. 58.1%, P = 0.120; 3-year OS: 83.3% vs. 75.3%, P = 0.155), and the adjusted analyses yielded similar findings. Exploratory subgroup analyses suggested a potential survival benefit among non-pCR patients with pathological downstaging (3-year DFS: 82.5% vs. 57.6%, P = 0.028; 3-year OS: 93.8% vs. 72.6%, P = 0.022). Further analysis showed no significant differences in 3-year DFS or OS between adjuvant immunotherapy alone and adjuvant immunochemotherapy among non-pCR patients with pathological downstaging (83.3% vs. 81.7%, P = 0.890; 91.7% vs. 94.7%, P = 0.988).

**Conclusions:**

Among patients with ESCC treated with NICT, immunotherapy-based adjuvant treatment did not improve survival in those who achieved pCR or in the overall non-pCR population. A potential benefit was observed in non-pCR patients with pathological downstaging. In this subgroup, adjuvant immunotherapy alone and adjuvant immunochemotherapy showed comparable efficacy.

## 1 Introduction

Esophageal squamous cell carcinoma (ESCC) is one of the malignant tumors with high morbidity and mortality worldwide (1). For locally advanced resectable ESCC, standard treatment includes surgery after neoadjuvant chemoradiotherapy or neoadjuvant chemotherapy to improve R0 resection rates and long-term survival (2, 3). In recent years, the role of immunotherapy in the comprehensive treatment of esophageal cancer has attracted much attention. Previous studies have shown that adding immunotherapy to the first-line treatment of advanced esophageal cancer can significantly improve efficacy (4–6). Recently, neoadjuvant immunochemotherapy (NICT) is increasingly used in the neoadjuvant treatment of locally advanced ESCC, with the aim of enhancing pathological response rates (7, 8).

Pathological complete response (pCR) is defined as the absence of residual tumor after neoadjuvant treatment and has been regarded as a marker of good prognosis (9). Patients who achieve pCR have a lower risk of recurrence and a longer disease-free survival. In theory, patients who achieve pCR may have been cured, but in reality a considerable proportion of patients with pCR still have recurrence after surgery (10). Previous studies have reported that about 20% of patients with pCR relapse within 2–3 years after surgery (11–13). Therefore, there is still debate about whether to adopt postoperative observation or adjuvant treatment strategies for patients who achieve pCR.

Conversely, for patients with non-pCR, the risk of recurrence is significantly increased (12, 13). The CheckMate577 clinical trial demonstrated that postoperative adjuvant nivolumab therapy can increase median disease-free survival from 11.0 months to 22.4 months in esophageal cancer patients with non-pCR after neoadjuvant chemoradiotherapy (14). This study established a new standard for postoperative immunotherapy in non-pCR patients following neoadjuvant chemoradiotherapy. However, the CheckMate 577 study population primarily received combined chemotherapy and radiotherapy. It remains unclear whether non-pCR patients after NICT should receive adjuvant immunotherapy. Furthermore, for these non-pCR patients, it is still uncertain whether adjuvant treatment should consist of immunotherapy alone or immunochemotherapy.

Therefore, we conducted a real-world retrospective study to assess the efficacy of immunotherapy-based adjuvant treatment after surgery in patients with ESCC who received NICT, stratified by pathological response status.

## 2 Materials and methods

### 2.1 Patients

This study included patients with locally advanced ESCC who received NICT and underwent radical resection at Anyang Tumor Hospital between 2019 and 2023. All patients met the following inclusion criteria: (1) ESCC confirmed by preoperative endoscopic biopsy; (2) clinical stage II–IVA disease; (3) receipt of NICT followed by radical surgical resection; and (4) clinical data were complete and traceable. Patients were excluded if they met any of the following criteria: (1) non-squamous esophageal carcinoma; (2) positive resection margins; (3) concomitant second primary malignancy; (4) receipt of adjuvant chemoradiotherapy; or (5) recurrence or death within 3 months after surgery. Finally, 260 patients met the inclusion criteria (**Supplementary Figure 1**). Staging followed the 8th AJCC/UICC TNM system (15). This study was approved by the Ethics Committee of Anyang Tumor Hospital (Approval No. 2023WZ29K01). Given the retrospective nature of the study, the requirement for informed consent was waived.

### 2.2 Treatment regimen

In this study, the NICT regimen consisted of two cycles of a programmed cell death protein 1 (PD-1) inhibitor combined with taxane- and platinum-based chemotherapy. Curative esophagectomy was performed 4–8 weeks following NICT completion, using either the McKeown or Ivor Lewis approach. To date, standardized guidelines remain lacking regarding adjuvant treatment for patients undergoing curative resection after NICT. Some patients received immunotherapy-based adjuvant treatment, including immunotherapy alone or immunochemotherapy, starting 4–8 weeks after surgery, whereas the remaining patients underwent postoperative observation only. Because no standard guideline currently defines the postoperative adjuvant treatment regimen, number of cycles, or treatment duration for patients with ESCC undergoing surgery after NICT, the number of adjuvant treatment cycles in this study was not predefined. Instead, it was determined according to postoperative recovery, physician judgment, patient tolerance, patient preference, compliance, and other real-world factors. Postoperative immunotherapy generally continued the same PD-1 inhibitor used during neoadjuvant treatment and was administered at the label-recommended dose, usually every 3 weeks, without dose reduction. For patients receiving adjuvant immunochemotherapy, the chemotherapy regimen consisted of nab-paclitaxel plus platinum. Details of the adjuvant treatment regimens are provided in **Supplementary Table 1**.

### 2.3 Pathological examination, endpoints and follow-up

Each patient’s pathological specimen was independently reviewed by two experienced pathologists. Pathological complete response was defined as the absence of viable tumor cells in the primary tumor and all resected lymph nodes. Pathological downstaging was defined as a decrease in T stage and/or N stage on pathological staging compared with baseline clinical staging. The primary endpoint was disease-free survival (DFS), calculated from the date of surgery until the earliest occurrence of tumor recurrence or death. The secondary endpoint was overall survival (OS), defined as the time from the date of surgery to death from any cause. Routine follow-up was conducted for all patients, involving imaging examinations every 3–6 months to assess for tumor recurrence or metastasis. The last follow-up was conducted in August 2025, and patients without events were censored at the time of their last follow-up.

### 2.4 Safety assessment

Safety outcomes were evaluated by reviewing outpatient and inpatient medical records, laboratory examinations, imaging reports and treatment records. Adverse events (AEs) were graded in accordance with CTCAE version 5.0. Given that there are no unified guidelines or predefined standard cycles for postoperative adjuvant therapy following NICT, treatment delays, interruptions, dose reductions and permanent treatment discontinuation were only defined as adverse event-related adjustments when explicitly documented to be caused by adverse events in medical records. Reduced treatment cycles resulting from patient preference, poor treatment compliance, economic factors, attending physician’s clinical judgment or unknown reasons were not classified as toxicity-associated treatment modifications.

### 2.5 Statistical analysis

Eligible patients were first stratified by postoperative pathological response status (pCR vs. non-pCR), and outcomes were compared between the immunotherapy-based adjuvant treatment group and the observation group within each stratum. Propensity score matching (PSM, 1:1 nearest-neighbor matching with a caliper of 0.2) and inverse probability of treatment weighting (IPTW) were used to balance the covariates between the immunotherapy-based adjuvant treatment group and the observation group (details provided in the footnotes of **Supplementary Tables 2, 3**). DFS and OS were estimated using the Kaplan-Meier method and compared with the log-rank test, with hazard ratios (HRs) and 95% confidence intervals (CIs) calculated using Cox proportional hazards models. Owing to the limited sample size, survival comparisons among the three groups (adjuvant immunotherapy alone vs. adjuvant immunochemotherapy vs. observation) were performed only in the unadjusted population.

Subgroup analysis was performed only in the non-pCR population, with subgroups defined by pathological lymph node status (ypN0 vs. ypN+) and pathological downstaging (yes vs. no). Treatment-by-subgroup interactions were assessed by including a multiplicative interaction term between treatment group and subgroup variable in the Cox proportional hazards model. All statistical analyses were performed using R software (version 4.5.2). All statistical tests were two-sided, and P value < 0.05 was considered statistically significant.

## 3 Results

### 3.1 Baseline characteristics

A total of 260 patients were included in this study. The median follow-up was 36.8 months. In the overall cohort, the median age was 69 years, and 165 patients (63.5%) were male. Based on pathological response, 84 patients (32.3%) achieved a pCR, whereas 176 patients (67.7%) did not. Regarding postoperative adjuvant therapy, 81 patients (31.2%) received immunotherapy-based adjuvant treatment, including 28 (10.8%) treated with immunotherapy alone and 53 (20.4%) treated with immunochemotherapy (**Table 1**). Among patients with pCR, 26 patients were in immunotherapy-based adjuvant treatment group and 58 patients were in observation group, whereas among patients with non-pCR, 55 patients were in immunotherapy-based adjuvant treatment group and 121 were in observation group. Detailed baseline characteristics within the pCR and non-pCR strata before and after PSM/IPTW adjustment are provided in **Supplementary Tables 2**, **3**. In the unadjusted analysis, significant baseline imbalances were observed between the immunotherapy-based adjuvant treatment group and observation group within both pathological response strata. After adjustment using PSM and IPTW, the distributions of covariates were effectively balanced between groups, with no statistically significant differences remaining (**Supplementary Tables 2**, **3**).

**Table 1:** Baseline clinicopathological characteristics of the overall cohort.

Characteristic	Total (N = 260)
Sex	
Male	165 (63.5%)
Female	95 (36.5%)
Age, years	69.0(63.0, 72.5)
ECOG performance status	
0	199 (76.5%)
1	61 (23.5%)
Tumor location	
Upper thoracic	60 (23.1%)
Middle thoracic	170 (65.4%)
Lower thoracic	30 (11.5%)
Tumor length, cm	5.00(4.00, 7.00)
Clinical T stage (cT)	
T2	23 (8.8%)
T3	214 (82.3%)
T4a	23 (8.8%)
Clinical N stage (cN)	
N0	45 (17.3%)
N1	170 (65.4%)
N2	40 (15.4%)
N3	5 (1.9%)
Clinical TNM stage (cTNM)	
Stage II	53 (20.4%)
Stage III	181 (69.6%)
Stage IVA	26 (10.0%)
Neoadjuvant immunotherapy agent	
Sintilimab	198 (76.2%)
Camrelizumab	38 (14.6%)
Penpulimab	17 (6.5%)
Pembrolizumab	7 (2.7%)
Neoadjuvant chemotherapy regimen	
Nab-paclitaxel + platinum	230 (88.5%)
Paclitaxel + platinum	30 (11.5%)
Surgical approach	
McKeown	219 (84.2%)
Ivor Lewis	41 (15.8%)
Pathological T stage (ypT)	
T0	87 (33.5%)
T1	56 (21.5%)
T2	51 (19.6%)
T3	66 (25.4%)
Pathological N stage (ypN)	
N0	194 (74.6%)
N1	37 (14.2%)
N2	27 (10.4%)
N3	2 (0.8%)
Pathological downstaging	
No	52 (20.0%)
Yes	208 (80.0%)
pCR	
No	176 (67.7%)
Yes	84 (32.3%)
Adjuvant treatment	
None	179 (68.8%)
Immunotherapy alone	28 (10.8%)
Immunochemotherapy	53 (20.4%)
The number of dissected lymph nodes	21.0 (16.0, 27.0)
Postoperative pathological grade	
No residual tumor	84 (32.3%)
Grade 1	20 (7.7%)
Grade 2	136 (52.3%)
Grade 3	20(7.7%)

Categorical variables are presented as n (%), and continuous variables as median(IQR). ECOG, Eastern Cooperative Oncology Group; TNM, tumor node metastasis; pCR, pathological complete response.

### 3.2 Survival outcomes stratified by pathological response

**Figure 1:**
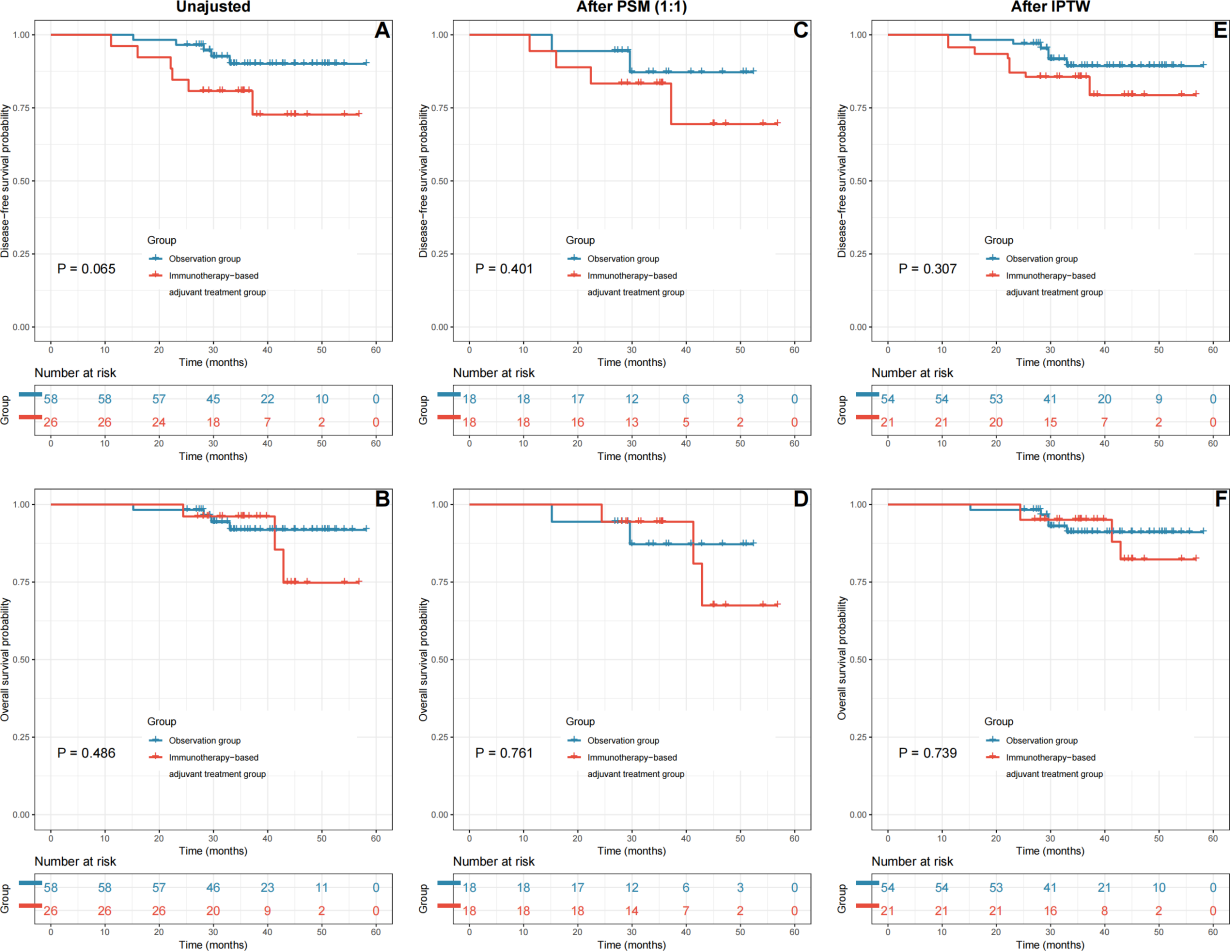
Kaplan–Meier survival curves comparing immunotherapy-based adjuvant treatment with observation for disease-free survival (DFS) and overall survival (OS) among patients achieving pCR. **(A)** DFS in the unadjusted analysis; **(B)** OS in the unadjusted analysis; **(C)** DFS in the propensity score–matched (PSM, 1:1) population; **(D)** OS in the PSM population; **(E)** DFS in the inverse probability of treatment weighting (IPTW)–weighted population; and **(F)** OS in the IPTW-weighted population.

Among patients with pCR, immunotherapy-based adjuvant treatment was not associated with improved survival outcomes compared with observation, with comparable 3-year DFS (80.8% vs. 90.1%, *P* = 0.065) and OS rates (96.2% vs. 91.8%, *P* = 0.486) in unadjusted analyses. These findings remained consistent after adjustment using PSM and IPTW (**Figure 1**, **Table 2**).

**Table 2:** Survival outcomes and univariable Cox proportional hazards analyses comparing immunotherapy-based adjuvant treatment with observation among patients with pCR.

Analysis method	Outcome	3-year DFS/OS rate (%) (Immunotherapy-based adjuvant treatment vs Observation)	HR (95% CI)	*P* value
Unadjusted	DFS	80.8% vs. 90.1%	2.90 (0.89–9.52)	0.078
	OS	96.2% vs. 91.8%	1.69 (0.38–7.56)	0.491
PSM	DFS	83.3% vs. 87.2%	2.04 (0.37–11.14)	0.410
	OS	94.4% vs. 87.2%	1.32 (0.22–7.92)	0.762
IPTW	DFS	85.6% vs. 89.3%	1.98 (0.53–7.34)	0.307
	OS	95.1% vs. 91.1%	1.31 (0.27–6.46)	0.739

DFS, disease-free survival; OS, overall survival; pCR, pathological complete response; PSM, propensity score matching; IPTW, inverse probability of treatment weighting; HR, hazard ratio; CI, confidence interval. Hazard ratios and P values were derived from univariable Cox proportional hazards regression analyses performed in unadjusted, matched, and weighted populations.

**Figure 2:**
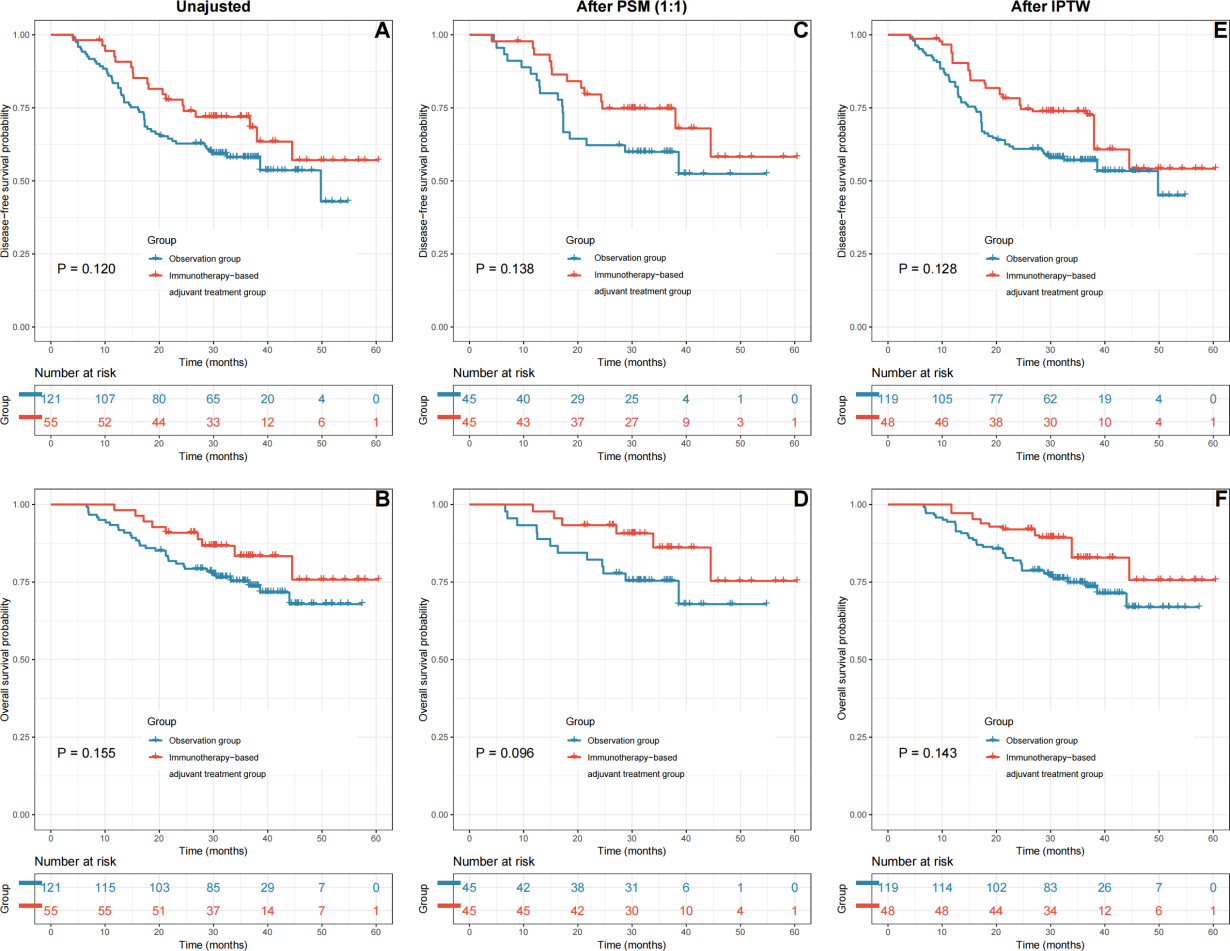
Kaplan–Meier survival curves comparing immunotherapy-based adjuvant treatment with observation for disease-free survival (DFS) and overall survival (OS) among patients with non-pCR. **(A)** DFS in the unadjusted analysis; **(B)** OS in the unadjusted analysis; **(C)** DFS in the propensity score–matched (PSM, 1:1) population; **(D)** OS in the PSM population; **(E)** DFS in the inverse probability of treatment weighting (IPTW)–weighted population; and **(F)** OS in the IPTW-weighted population.

Similarly, among patients with non-pCR, immunotherapy-based adjuvant treatment did not confer a statistically significant survival advantage over observation, with comparable 3-year DFS (71.9% vs. 58.1%, *P* = 0.120) and OS (83.3% vs. 75.3%, *P* = 0.155) rates, and the results remained robust after PSM and IPTW adjustment (**Figure 2**; **Table 3**).

**Table 3:** Survival outcomes and univariable Cox proportional hazards analyses comparing immunotherapy-based adjuvant treatment with observation among patients with non-pCR.

Analysis method	Outcome	3-year DFS/OS rate (%) (Immunotherapy-based adjuvant treatment vs Observation)	HR (95% CI)	*P* value
Unadjusted	DFS	71.9% vs. 58.1%	0.66 (0.38–1.12)	0.123
	OS	83.3% vs. 75.3%	0.59 (0.28–1.23)	0.160
PSM	DFS	74.8% vs. 59.9%	0.59 (0.29–1.19)	0.141
	OS	86.1% vs. 75.4%	0.44 (0.17–1.19)	0.105
IPTW	DFS	73.8% vs. 57.1%	0.64 (0.36–1.14)	0.128
	OS	82.9% vs. 74.9%	0.55 (0.25–1.23)	0.143

DFS, disease-free survival; OS, overall survival; pCR, pathological complete response; PSM, propensity score matching; IPTW, inverse probability of treatment weighting; HR, hazard ratio; CI, confidence interval. Hazard ratios and P values were derived from univariable Cox proportional hazards regression analyses performed in unadjusted, matched, and weighted populations.

We conducted Kaplan-Meier survival analysis to compare survival outcomes among the observation, adjuvant immunotherapy alone, and adjuvant immunochemotherapy groups. In both pCR and non-pCR populations, unadjusted comparisons showed no significant differences in DFS or OS among the three groups (pCR population: DFS, *P* = 0.183; OS, *P* = 0.650; non-pCR population: DFS, *P* = 0.301; OS, *P* = 0.348) (**Supplementary Figures 2**, **3**).

### 3.3 Subgroup analysis within the non-pCR population

**Figure 3:**
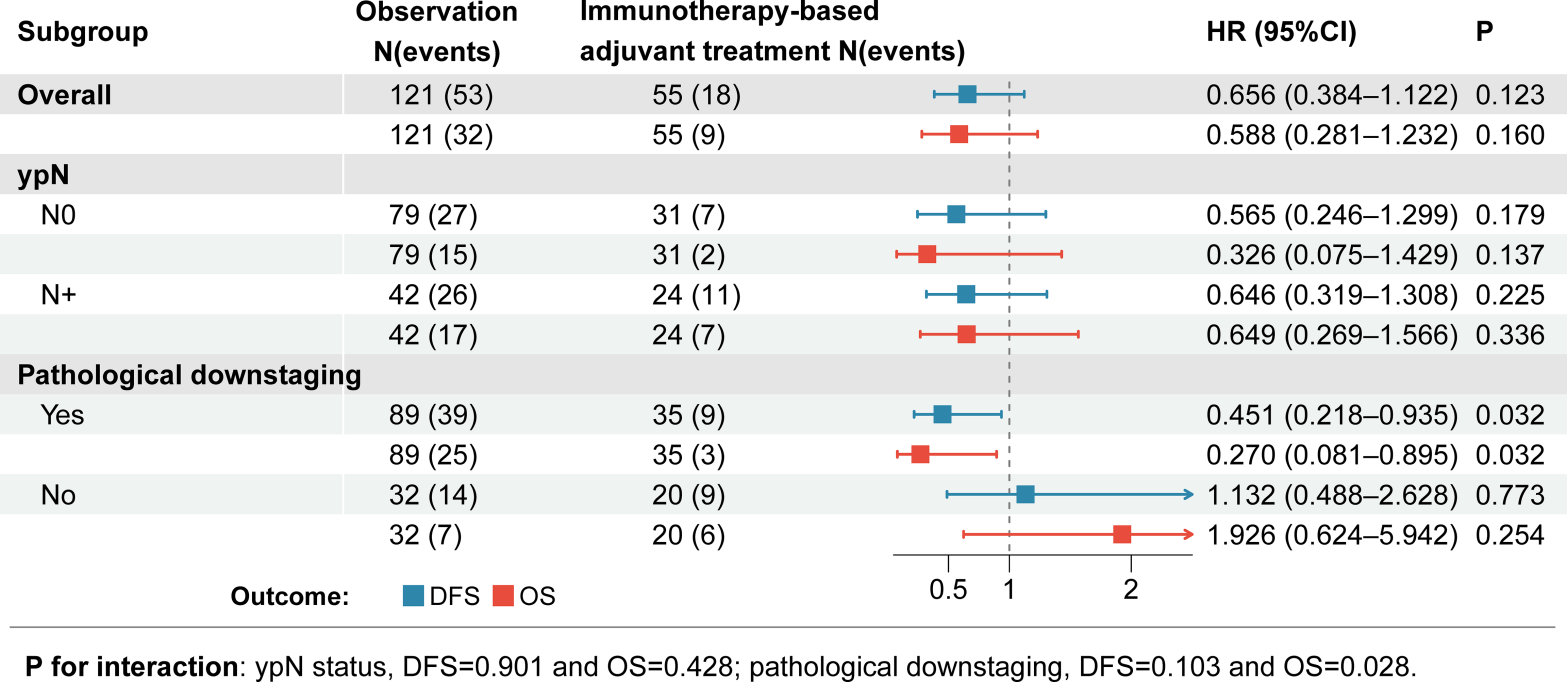
Subgroup analysis of survival outcomes associated with immunotherapy-based adjuvant treatment among patients with non-pCR. Forest plot showing hazard ratios (HRs) and 95% confidence intervals (CIs) for disease-free survival (DFS) and overall survival (OS) comparing immunotherapy-based adjuvant treatment with observation across prespecified subgroups, including pathological lymph node status (ypN0 vs. ypN+) and pathological downstaging (yes vs. no). The vertical dashed line indicates an HR of 1. Numbers of patients and events are shown for each subgroup. Blue markers represent DFS, and red markers represent OS.

**Figure 4:**
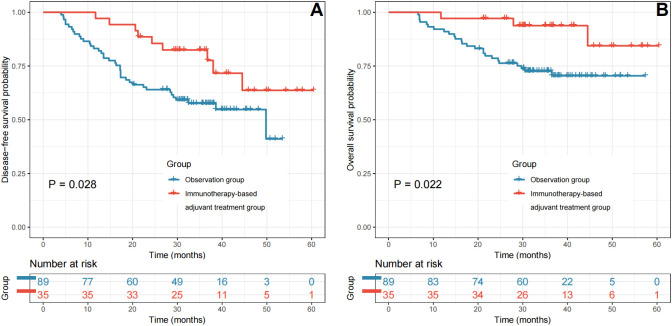
Kaplan–Meier survival curves comparing immunotherapy-based adjuvant treatment with observation for disease-free survival **(A)** and overall survival **(B)** among patients with pathological downstaging in the non-pCR population.

Within the non-pCR population, prespecified subgroup analyses were performed according to ypN status and pathological downstaging. No clear survival benefit of immunotherapy-based adjuvant treatment was observed in the ypN0, ypN+, or non-downstaging subgroups. In contrast, among patients with pathological downstaging, immunotherapy-based adjuvant treatment was associated with more favorable DFS (*HR*, 0.45; 95% *CI*, 0.22-0.94; *P* = 0.032) and OS (*HR*, 0.27; 95% *CI*, 0.08-0.90; *P* = 0.032). However, interaction testing showed that the treatment-by-downstaging interaction was not statistically significant for DFS (*P* for interaction=0.103) but was statistically significant for OS (*P* for interaction=0.028) (**Figure 3**). Kaplan–Meier analyses further showed that, in non-pCR patients with pathological downstaging, immunotherapy-based adjuvant treatment was associated with significantly improved 3-year DFS (82.5% vs. 57.6%, *P* = 0.028) and OS (93.8% vs. 72.6%, *P* = 0.022) compared with observation (**Figure 4**). Among these patients, survival outcomes were comparable between adjuvant immunotherapy alone and adjuvant immunochemotherapy in terms of both 3-year DFS (83.3% vs. 81.7%, *P* = 0.890) and OS (91.7% vs. 94.7%, *P* = 0.988) (**Supplementary Figure 4**).

### 3.4 Patterns of recurrence and metastasis

During follow-up, 67 patients in the overall population experienced recurrence. Specifically, 36 patients (53.7%) developed locoregional recurrence, 18 (26.9%) developed distant metastasis, and 13 (19.4%) developed both locoregional recurrence and distant metastasis. The sites of recurrence and metastasis were as follows: mediastinal lymph nodes (n = 45), lung (n = 11), cervical lymph nodes and anastomosis (n = 9 each), supraclavicular lymph nodes (n = 5), celiac lymph nodes (n = 4), adrenal glands, chest wall, liver, and bone (n = 2 each), and pleura (n = 1).

### 3.5 Safety outcomes

Safety outcomes are summarized in **Supplementary Table 4**. Any-grade immune-related adverse events occurred more frequently in the immunotherapy-based adjuvant treatment group than in the observation group (21.0% vs. 2.2%, P<0.001), whereas Grade ≥3 immune-related adverse events occurred in 3.7% and 0% of patients, respectively (P = 0.029). Among patients receiving adjuvant immunochemotherapy, any-grade and Grade ≥3 chemotherapy-related toxicities occurred in 60.4% and 15.1% of patients, respectively. In the overall adjuvant treatment group, AE-related treatment interruption/delay and permanent immunotherapy discontinuation occurred in 12.3% and 3.7% of patients, respectively. Among patients receiving adjuvant immunochemotherapy, chemotherapy dose reduction and permanent chemotherapy discontinuation occurred in 9.4% and 5.7% of patients, respectively. No treatment-related deaths were observed.

## 4 Discussion

Based on real-world, single-center data, this study investigated postoperative adjuvant treatment strategies for patients with ESCC after NICT, with a focus on pathological response status. The main findings were as follows: among patients achieving pCR, postoperative immunotherapy-based adjuvant treatment provided no survival benefit compared with observation. In contrast, in the non-pCR population, the benefit of immunotherapy-based adjuvant treatment was markedly heterogeneous, with significant improvements in DFS and OS observed only in patients who achieved pathological downstaging. Furthermore, within the benefiting subgroup, immunotherapy alone demonstrated efficacy comparable to that of immunochemotherapy, providing evidence to inform optimization of adjuvant treatment regimens.

For patients who achieved pCR, our findings support a de-escalation strategy. Despite the theoretical risk of occult metastasis, both PSM and IPTW analyses consistently showed that immunotherapy-based adjuvant treatment did not improve DFS and OS. Two retrospective studies also found that patients with esophageal cancer who achieved pCR after NICT had a good prognosis, and adjuvant immunotherapy did not significantly improve patient survival outcomes compared with no adjuvant treatment (16, 17). From an immunologic perspective, pCR means that tumors in primary and regional lymph nodes have been completely cleared, and NICT may have successfully induced long-term tumor-specific T-cell memory (18). In this state, the continued use of immune checkpoint inhibitors lacks targets, which not only has no additional survival benefit, but also increases the risk of immune-related adverse events and medical financial burden (19). Therefore, for patients who achieve pCR after NICT, avoiding overtreatment should be a priority clinical decision.

In contrast, the recurrence rate of non-pCR patients is significantly increased, making this group a key target for improving prognosis. The landmark CheckMate577 trial established nivolumab’s status as standard adjuvant treatment in patients with non-pCR after neoadjuvant chemoradiotherapy (14). However, Xie et al. conducted a PSM analysis of 155 ESCC patients undergoing NICT followed by surgery and found that in the non-pCR population, adjuvant treatment did not bring survival benefits (20). Our study also found that the overall non-pCR population after NICT did not achieve statistically significant survival benefits from immunotherapy-based adjuvant treatment. This difference from CheckMate577 results may be attributed to a different induction treatment model. Radiotherapy has an *in situ* vaccine effect, reshaping the tumor microenvironment and making residual lesions sensitive to subsequent immunotherapy (21, 22). Therefore, simply extrapolating the conclusions of CheckMate577 to the NICT population may not be appropriate.

As immunotherapy-based adjuvant treatment did not significantly improve survival in the overall non-pCR population, identifying potential benefit subgroups is clinically important. This study identified a key subgroup that may benefit among non-pCR patients: those with pathological downstaging. This subgroup indicates that the tumor remains somewhat sensitive to prior treatment, and both DFS and OS are significantly improved after receiving immunotherapy-based adjuvant treatment. A potential mechanism is that pathological downstaging suggests partial responsiveness of the tumor to immune checkpoint inhibitors, and the presence of residual disease is more likely related to insufficient treatment exposure rather than complete drug resistance. In this setting, immunotherapy-based adjuvant treatment is likely to work by clearing minimal residual disease (23). On the contrary, tumors in patients without pathological downstaging may have an “immune desert” phenotype, and it is difficult to reverse drug resistance by simply extending treatment with similar drugs (24, 25). In the future, for such refractory subgroups, it may be necessary to explore other treatment options with more aggressive or different mechanisms, such as adjuvant radiotherapy, targeted therapy, or new drugs in clinical trials.

Although immunotherapy shows promise in the adjuvant field, many practical questions remain to be answered. As a type of systemic therapy, immunotherapy has already played a role in the neoadjuvant phase. How long it will continue to be given after surgery and whether a single drug is sufficient are still unknown areas. Our results demonstrated that in the non-pCR population with pathological downstaging, immunotherapy alone achieved favorable efficacy, and the addition of chemotherapy showed no significant additional benefit.

This single-center retrospective real-world study has several limitations. First, postoperative adjuvant treatment was not randomly assigned, leading to potential selection bias and confounding by indication. Although PSM and IPTW balanced measured covariates, unmeasured factors such as physicians’ subjective judgment, patient compliance, and economic status could not be fully addressed. Second, postoperative immunotherapy alone and immunochemotherapy were combined in the primary analysis, and the limited sample size precluded adjusted comparisons between these strategies. Third, the subgroup analyses were limited by small sample sizes and few events, resulting in insufficient statistical power and wide confidence intervals. Therefore, the subgroup findings should be interpreted as exploratory rather than definitive evidence of treatment-effect modification, and validation in larger prospective studies is needed. Finally, detailed post-recurrence treatment data were incomplete for some patients, particularly those treated outside our center. Potential crossover to immunotherapy or other effective systemic therapies after recurrence may have attenuated OS differences and underestimated the potential OS benefit of adjuvant treatment. Nonetheless, this study still has important clinical implications. In the context of widespread use of neoadjuvant immunotherapy, this study provides valuable real-world evidence for formulating postoperative adjuvant therapy strategies based on pathological response.

In summary, this study proposes an adjuvant treatment strategy for esophageal squamous cell carcinoma based on pathological response. Observation with surveillance is recommended for patients achieving pCR after NICT to avoid overtreatment. For non-pCR patients, an exploratory benefit signal of immunotherapy-based adjuvant treatment was observed among those achieving pathological downstaging, in whom immunotherapy alone may be sufficient. In patients without downstaging, routine immunotherapy-based adjuvant treatment shows limited benefit, highlighting the need for novel therapeutic strategies.

## Data Availability

The raw data supporting the conclusions of this article will be made available by the authors, without undue reservation.
