# Supplementary material for: Immunotherapy-based adjuvant treatment after neoadjuvant immunochemotherapy in esophageal squamous cell carcinoma according to pathological response
Source: Front Immunol. 2026 Jun 3;17:1802037. doi: 10.3389/fimmu.2026.1802037 (PMC13272288; doi:10.3389/fimmu.2026.1802037)
Supplement: Supplementary file 1 [file Table1.docx]

Table S1.Postoperative adjuvant regimen and treatment exposure in the immunotherapy-based adjuvant treatment group.

| **Regimen component** | **Immunotherapy-based adjuvant treatment group (n=81)** |
| --- | --- |
| **Treatment timing and principle** |  |
| Timing of postoperative adjuvant treatment initiation | 4–8 weeks after surgery after adequate postoperative recovery |
| Treatment decision | Determined by postoperative recovery, pathological response, patient preference, treating physicians' clinical judgment and other real-world factors |
| **Postoperative immunotherapy** |  |
| General immunotherapy principle | Usually continued the same PD-1 inhibitor used during neoadjuvant treatment; administered at label-recommended fixed doses without dose reduction, generally 200 mg intravenously every 3 weeks |
| PD-1 inhibitor used | Sintilimab: 50 (61.7%); camrelizumab: 15 (18.5%); penpulimab: 10 (12.3%); pembrolizumab: 6 (7.4%) |
| Immunotherapy cycles, median (IQR) | 2 (1–4) |
| Immunotherapy cycles, range | 1–24 |
| 1–2 immunotherapy cycles | 51 (63.0%) |
| 3–4 immunotherapy cycles | 19 (23.5%) |
| ≥5 immunotherapy cycles | 11 (13.6%) |
| **Postoperative chemotherapy among patients receiving immunochemotherapy** |  |
| Chemotherapy regimen | Nab-paclitaxel plus platinum |
| Nab-paclitaxel plus cisplatin | 26/53 (49.1%) |
| Nab-paclitaxel plus nedaplatin | 27/53 (50.9%) |
| Chemotherapy cycles, median (IQR) | 2 (2–3) |
| Chemotherapy cycles, range | 1–4 |
| 1–2 chemotherapy cycles | 37/53 (69.8%) |
| 3–4 chemotherapy cycles | 16/53 (30.2%) |

Note: Values are presented as n (%) or median (IQR). IQR, interquartile range; PD-1, programmed cell death protein 1. The denominator for chemotherapy-related rows is 53, because only patients who received adjuvant immunochemotherapy were exposed to postoperative chemotherapy.

Table S2. Baseline characteristics among patients with pCR.

| Characteristic | Unadjusted | | | PSM (1:1) | | | IPTW | | |
| --- | --- | --- | --- | --- | --- | --- | --- | --- | --- |
|  | Observation group(n=58) | Immunotherapy-based adjuvant treatment group(n=26) | *P* value | Observation group(n=18) | Immunotherapy-based adjuvant treatment group(n=18) | *P* value | Observation group(n=53.5) | Immunotherapy-based adjuvant treatment group(n=21.2) | *P* value |
| Sex |  |  | 0.146 |  |  | 1.000 |  |  | 0.719 |
| Male | 31 (53.4%) | 19 (73.1%) |  | 15 (83.3%) | 14 (77.8%) |  | 32.0 (59.9%) | 13.8 (65.1%) |  |
| Female | 27 (46.6%) | 7 (26.9%) |  | 3 (16.7%) | 4 (22.2%) |  | 21.5 (40.1%) | 7.4 (34.9%) |  |
| Age, years | 69.0 (63.3, 73.0) | 63.0 (59.3, 68.5) | 0.007 | 67.0 (61.3, 70.0) | 63.0 (61.2, 69.8) | 0.600 | 68.3 (61.8, 72.0) | 63.0 (61.2, 72.0) | 0.474 |
| ECOG performance status |  |  | 0.538 |  |  | 1.000 |  |  | 0.943 |
| 0 | 44 (75.9%) | 22 (84.6%) |  | 16 (88.9%) | 16 (88.9%) |  | 42.4 (79.3%) | 16.6 (78.4%) |  |
| 1 | 14 (24.1%) | 4 (15.4%) |  | 2 (11.1%) | 2 (11.1%) |  | 11.1 (20.7%) | 4.6 (21.6%) |  |
| Tumor location |  |  | 0.700 |  |  | 0.890 |  |  | 0.886 |
| Upper thoracic | 12 (20.7%) | 7 (26.9%) |  | 5 (27.8%) | 6 (33.3%) |  | 11.9 (22.2%) | 4.1 (19.2%) |  |
| Middle thoracic | 39 (67.2%) | 15 (57.7%) |  | 9 (50.0%) | 9 (50.0%) |  | 34.1 (63.7%) | 13.2 (62.3%) |  |
| Lower thoracic | 7 (12.1%) | 4 (15.4%) |  | 4 (22.2%) | 3 (16.7%) |  | 7.6 (14.2%) | 3.9 (18.4%) |  |
| Tumor length, cm | 5.00 (4.00, 6.75) | 6.00 (5.00, 6.88) | 0.321 | 6.00 (5.00, 7.00) | 6.00 (5.00, 7.00) | 0.543 | 5.00 (4.00, 7.00) | 6.00 (4.41, 7.00) | 0.456 |
| Clinical T stage (cT) |  |  | 0.318 |  |  | 0.513 |  |  | 0.472 |
| T2 | 11 (19.0%) | 2 (7.7%) |  | 0 (0.0%) | 1 (5.6%) |  | 8.1 (15.2%) | 1.2 (5.7%) |  |
| T3 | 42 (72.4%) | 20 (76.9%) |  | 16 (88.9%) | 14 (77.8%) |  | 40.2 (75.1%) | 17.7 (83.5%) |  |
| T4a | 5 (8.6%) | 4 (15.4%) |  | 2 (11.1%) | 3 (16.7%) |  | 5.2 (9.7%) | 2.3 (10.8%) |  |
| Clinical N stage (cN) |  |  | 0.730 |  |  | 0.946 |  |  | 0.941 |
| N0 | 12 (20.7%) | 3 (11.5%) |  | 2 (11.1%) | 2 (11.1%) |  | 9.6 (17.9%) | 2.6 (12.5%) |  |
| N1 | 37 (63.8%) | 18 (69.2%) |  | 14 (77.8%) | 13 (72.2%) |  | 36.0 (67.2%) | 15.3 (72.4%) |  |
| N2 | 8 (13.8%) | 4 (15.4%) |  | 1 (5.6%) | 2 (11.1%) |  | 6.9 (12.9%) | 2.7 (13.0%) |  |
| N3 | 1 (1.7%) | 1 (3.8%) |  | 1 (5.6%) | 1 (5.6%) |  | 1.0 (1.9%) | 0.5 (2.2%) |  |
| Clinical TNM stage (cTNM) |  |  | 0.513 |  |  | 0.887 |  |  | 0.832 |
| Stage II | 15 (25.9%) | 4 (15.4%) |  | 2 (11.1%) | 3 (16.7%) |  | 11.8 (22.0%) | 3.5 (16.3%) |  |
| Stage III | 37 (63.8%) | 18 (69.2%) |  | 13 (72.2%) | 12 (66.7%) |  | 35.5 (66.4%) | 15.4 (72.9%) |  |
| Stage IVA | 6 (10.3%) | 4 (15.4%) |  | 3 (16.7%) | 3 (16.7%) |  | 6.2 (11.6%) | 2.3 (10.8%) |  |
| Neoadjuvant immunotherapy agent |  |  | 0.001 |  |  | 0.914 |  |  | 0.184 |
| Sintilimab | 48 (82.8%) | 15 (57.7%) |  | 13 (72.2%) | 14 (77.8%) |  | 43.5 (81.2%) | 15.4 (72.7%) |  |
| Camrelizumab | 9 (15.5%) | 3 (11.5%) |  | 4 (22.2%) | 3 (16.7%) |  | 9.0 (16.9%) | 3.2 (14.9%) |  |
| Pembrolizumab | 0 (0.0%) | 2 (7.7%) |  | 0 (0.0%) | 0 (0.0%) |  | 0.0 (0.0%) | 0.6 (2.9%) |  |
| Penpulimab | 1 (1.7%) | 6 (23.1%) |  | 1 (5.6%) | 1 (5.6%) |  | 1.0 (1.9%) | 2.0 (9.5%) |  |
| Neoadjuvant chemotherapy regimen |  |  | 0.444 |  |  | 1.000 |  |  | 0.799 |
| Nab-paclitaxel + platinum | 52 (89.7%) | 21 (80.8%) |  | 17 (94.4%) | 16 (88.9%) |  | 48 (89.1%) | 18.6 (87.9%) |  |
| Paclitaxel + platinum | 6 (10.3%) | 5 (19.2%) |  | 1 (5.6%) | 2 (11.1%) |  | 5.5 (10.2%) | 2.6 (12.1%) |  |
| Surgical approach |  |  | 1.000 |  |  | 0.688 |  |  | 0.954 |
| McKeown | 46 (79.3%) | 21 (80.8%) |  | 13 (72.2%) | 15 (83.3%) |  | 42.8 (79.9%) | 17.1 (80.5%) |  |
| Ivor Lewis | 12 (20.7%) | 5 (19.2%) |  | 5(27.8%) | 3(16.7%) |  | 10.7 (20.1%) | 4.1 (19.5%) |  |
| The number of dissected lymph nodes | 21.0(15.3, 27.0) | 19.5(17.0, 23.8) | 0.977 | 21.0 (17.0, 31.5) | 19.5 (17.0, 23.8) | 0.646 | 20.6 (15.1, 27.0) | 19.6 (16.4, 22.9) | 0.862 |

Note: Categorical variables are presented as n (%), and continuous variables as median(IQR). ECOG, Eastern Cooperative Oncology Group; TNM, tumor node metastasis; pCR, pathologic complete response; PSM, propensity score matching; IPTW, inverse probability of treatment weighting. Propensity scores were estimated using logistic regression with the following covariates: age, sex, ECOG performance status, tumor location, tumor length, clinical T stage, clinical N stage, neoadjuvant immunotherapy agent, neoadjuvant chemotherapy regimen, surgical approach, and number of dissected lymph nodes. PSM used 1:1 nearest-neighbor matching without replacement on the logit(PS) scale with a caliper of 0.2. IPTW used stabilized inverse probability of treatment weights.

Table S3. Baseline characteristics among patients with non-pCR.

| Characteristic | Unadjusted | | | PSM (1:1) | | | IPTW | | |
| --- | --- | --- | --- | --- | --- | --- | --- | --- | --- |
|  | Observation group(n=121) | Immunotherapy-based adjuvant treatment group(n=55) | *P* value | Observation group(n=45) | Immunotherapy-based adjuvant treatment group(n=45) | *P* value | Observation group(n=118.9) | Immunotherapy-based adjuvant treatment group(n=47.6) | *P* value |
| Sex |  |  | 0.028 |  |  | 1.000 |  |  | 0.535 |
| Female | 35 (28.9%) | 26 (47.3%) |  | 19 (42.2%) | 19 (42.2%) |  | 41.9 (35.3%) | 19.4 (40.8%) |  |
| Male | 86 (71.1%) | 29 (52.7%) |  | 26 (57.8%) | 26 (57.8%) |  | 77.0 (64.7%) | 28.2 (59.2%) |  |
| Age, years | 69.0 (65.0, 72.0) | 70.0 (65.0, 74.0) | 0.341 | 70.0 (67.0, 74.0) | 70.0 (64.0, 73.0) | 0.749 | 69.0 (65.0, 73.0) | 69.0 (63.6, 72.9) | 0.956 |
| ECOG performance status |  |  | 0.463 |  |  | 1.000 |  |  | 0.982 |
| 0 | 89 (73.6%) | 44 (80.0%) |  | 36 (80.0%) | 35 (77.8%) |  | 87.8 (73.4%) | 35.2 (74.1%) |  |
| 1 | 32 (26.4%) | 11 (20.0%) |  | 9 (20.0%) | 10 (22.2%) |  | 31.1 (26.1%) | 12.4 (25.9%) |  |
| Tumor location |  |  | 0.268 |  |  | 0.770 |  |  | 0.973 |
| Upper thoracic | 24 (19.8%) | 17 (30.9%) |  | 11 (24.4%) | 14 (31.1%) |  | 27.9 (23.4%) | 11.4 (23.9%) |  |
| Middle thoracic | 83 (68.6%) | 33 (60.0%) |  | 29 (64.4%) | 26 (57.8%) |  | 78.3 (65.9%) | 31.7 (66.6%) |  |
| Lower thoracic | 14 (11.6%) | 5 (9.1%) |  | 5 (11.1%) | 5 (11.1%) |  | 12.7 (10.7%) | 4.5 (9.5%) |  |
| Tumor length, cm | 5.00 (4.00, 7.00) | 5.00 (4.00, 6.00) | 0.143 | 5.00 (4.00, 6.00) | 5.00 (5.00, 6.00) | 0.440 | 5.00 (4.00, 7.00) | 5.00 (4.02, 6.00) | 0.928 |
| Clinical T stage (cT) |  |  | 0.124 |  |  | 1.000 |  |  | 0.143 |
| T2 | 7 (5.8%) | 3 (5.5%) |  | 1 (2.2%) | 2 (4.4%) |  | 6.7 (5.6%) | 2.9 (6.2%) |  |
| T3 | 101 (83.5%) | 51 (92.7%) |  | 44 (97.8%) | 43 (95.6%) |  | 103.3 (86.9%) | 44.4 (93.2%) |  |
| T4a | 13 (10.7%) | 1 (1.8%) |  | 0 (0.0%) | 0 (0.0%) |  | 8.9 (7.5%) | 0.3 (0.7%) |  |
| Clinical N stage (cN) |  |  | 0.904 |  |  | 1.000 |  |  | 0.924 |
| N0 | 22 (18.2%) | 8 (14.5%) |  | 8 (17.8%) | 8 (17.8%) |  | 21.8 (18.3%) | 7.9 (16.5%) |  |
| N1 | 77 (63.6%) | 38 (69.1%) |  | 32 (71.1%) | 32 (71.1%) |  | 76.6 (64.4%) | 32.8 (68.8%) |  |
| N2 | 20 (16.5%) | 8 (14.5%) |  | 5 (11.1%) | 5 (11.1%) |  | 19.1 (16.1%) | 6.7 (14.0%) |  |
| N3 | 2 (1.7%) | 1 (1.8%) |  | 0 (0.0%) | 0 (0.0%) |  | 1.4 (1.2%) | 0.3 (0.7%) |  |
| Clinical TNM stage (cTNM) |  |  | 0.236 |  |  | 0.792 |  |  | 0.168 |
| Stage II | 23 (19.0%) | 11 (20.0%) |  | 8 (17.8%) | 10 (22.2%) |  | 23.3 (19.6%) | 10.8 (22.7%) |  |
| Stage III | 84 (69.4%) | 42 (76.4%) |  | 37 (82.2%) | 35 (77.8%) |  | 86.0 (72.3%) | 36.2 (76.0%) |  |
| Stage IVA | 14 (11.6%) | 2 (3.6%) |  | 0 (0.0%) | 0 (0.0%) |  | 9.6 (8.1%) | 0.6 (1.3%) |  |
| Neoadjuvant immunotherapy agent |  |  | 0.016 |  |  | 0.969 |  |  | 0.925 |
| Sintilimab | 100 (82.6%) | 35 (63.6%) |  | 32 (71.1%) | 32 (71.1%) |  | 91.8 (77.2%) | 34.5 (72.4%) |  |
| Camrelizumab | 14 (11.6%) | 12 (21.8%) |  | 10 (22.2%) | 9 (20.0%) |  | 18.4 (15.5%) | 9.0 (18.9%) |  |
| Pembrolizumab | 1 (0.8%) | 4 (7.3%) |  | 1 (2.2%) | 1 (2.2%) |  | 2.7 (2.3%) | 1.6 (3.4%) |  |
| Penpulimab | 6 (5.0%) | 4 (7.3%) |  | 2 (4.4%) | 3 (6.7%) |  | 6.0 (5.1%) | 2.5 (5.3%) |  |
| Neoadjuvant chemotherapy regimen |  |  | 0.451 |  |  | 0.673 |  |  | 0.995 |
| Nab-paclitaxel + platinum | 106 (87.6%) | 51 (92.7%) |  | 43 (95.6%) | 41 (91.1%) |  | 105.9 (89.1%) | 42.4 (89%) |  |
| Paclitaxel + platinum | 15 (12.4%) | 4 (7.3%) |  | 2 (4.4%) | 4 (8.9%) |  | 13.0 (10.9%) | 5.2 (11.0%) |  |
| Surgical approach |  |  | 1.000 |  |  | 0.737 |  |  | 0.934 |
| McKeown | 105 (86.8%) | 47 (85.5%) |  | 41 (91.1%) | 39 (86.7%) |  | 101.6 (85.4%) | 40.4 (84.9%) |  |
| Ivor Lewis | 16 (13.2%) | 8 (14.5%) |  | 4 (8.9%) | 6(13.3%) |  | 17.3 (14.6%) | 7.2 (16.1%) |  |
| Pathologic T stage (ypT) |  |  | 0.598 |  |  | 0.788 |  |  | 0.932 |
| T0 | 1 (0.8%) | 2 (3.6%) |  | 0 (0.0%) | 0 (0.0%) |  | 0.7 (0.6%) | 0.6 (1.3%) |  |
| T1 | 38 (31.4%) | 18 (32.7%) |  | 15 (33.3%) | 16 (35.6%) |  | 38.1 (32.0%) | 16.4 (34.4%) |  |
| T2 | 36 (29.8%) | 15 (27.3%) |  | 16 (35.6%) | 13 (28.9%) |  | 35.3 (29.7%) | 13.7 (28.8%) |  |
| T3 | 46 (38.0%) | 20 (36.4%) |  | 14 (31.1%) | 16 (35.6%) |  | 44.9 (37.7%) | 16.9 (35.5%) |  |
| Pathologic N stage (ypN) |  |  | 0.141 |  |  | 0.968 |  |  | 0.847 |
| N0 | 79 (65.3%) | 31 (56.4%) |  | 29 (64.4%) | 28 (62.2%) |  | 75.2 (63.2%) | 28.8 (60.6%) |  |
| N1 | 20 (16.5%) | 17 (30.9%) |  | 10 (22.2%) | 11 (24.4%) |  | 23.0 (19.3%) | 10.8 (22.7%) |  |
| N2 | 20 (16.5%) | 7 (12.7%) |  | 6 (13.3%) | 6 (13.3%) |  | 19.3 (16.3%) | 8.0 (16.8%) |  |
| N3 | 2 (1.7%) | 0 (0.0%) |  | 0 (0.0%) | 0 (0.0%) |  | 1.4 (1.2%) | 0.0 (0.0%) |  |
| Pathological downstaging |  |  | 0.247 |  |  | 0.367 |  |  | 0.458 |
| No | 32 (26.4%) | 20 (36.4%) |  | 12 (26.7%) | 17 (37.8%) |  | 37.1 (30.5%) | 17.6 (37.0%) |  |
| Yes | 89 (73.6%) | 35 (63.6%) |  | 33 (73.3%) | 28 (62.2%) |  | 82.7 (69.5%) | 30.0 (63.0%) |  |
| The number of dissected lymph nodes | 21.0 (16.0, 27.0) | 23.0 (17.0, 29.0) | 0.169 | 20.0 (17.0, 27.0) | 23.0 (18.0, 29.0) | 0.307 | 21.0 (17.0, 27.0) | 23.0 (17.0, 29.0) | 0.407 |
| Postoperative pathological grade |  |  | 0.844 |  |  | 0.920 |  |  | 0.879 |
| Grade 1 | 13 (10.7%) | 7 (12.7%) |  | 6 (13.3%) | 7 (15.6%) |  | 13.7 (11.5%) | 5.5 (11.6%) |  |
| Grade 2 | 95 (78.5%) | 41 (74.5%) |  | 33 (73.3%) | 33 (73.3%) |  | 91.4 (76.9%) | 35.1 (73.7%) |  |
| Grade 3 | 13 (10.7%) | 7 (12.7%) |  | 6 (13.3%) | 5 (11.1%) |  | 13.8 (11.6%) | 7.0 (14.7%) |  |

Note: Categorical variables are presented as n (%), and continuous variables as median(IQR). ECOG ,Eastern Cooperative Oncology Group ; TNM, tumor node metastasis; pCR, pathologic complete response; PSM, propensity score matching; IPTW, inverse probability of treatment weighting. Propensity scores were estimated using logistic regression with the following covariates: age, sex, ECOG performance status, tumor location, tumor length, clinical T stage, clinical N stage, neoadjuvant immunotherapy agent, neoadjuvant chemotherapy regimen, surgical approach, and number of dissected lymph nodes, pathologic T stage, pathologic N stage , postoperative pathological grade and pathological downstaging status. PSM used 1:1 nearest-neighbor matching without replacement on the logit(PS) scale with a caliper of 0.2. IPTW used stabilized inverse probability of treatment weights.

Table S4. Safety outcomes during the postoperative adjuvant/observation period.

| Safety outcome | Observation group (n=179) | Adjuvant immunotherapy alone group (n=28) | Adjuvant immunochemotherapy group (n=53) | Immunotherapy-based adjuvant treatment group (n=81) | P value |
| --- | --- | --- | --- | --- | --- |
| Any-grade immune-related adverse event | 4 (2.2%) | 7 (25.0%) | 10 (18.9%) | 17 (21.0%) | <0.001 |
| Grade ≥3 immune-related adverse event | 0 (0.0%) | 1 (3.6%) | 2 (3.8%) | 3 (3.7%) | 0.029 |
| Grade ≥3 immune-related pneumonitis | 0 (0.0%) | 1 (3.6%) | 0 (0.0%) | 1 (1.2%) | 0.312 |
| Grade ≥3 rash/pruritus | 0 (0.0%) | 0 (0.0%) | 1 (1.9%) | 1 (1.2%) | 0.312 |
| Grade ≥3 thyroid dysfunction | 0 (0.0%) | 0 (0.0%) | 0 (0.0%) | 0 (0.0%) | 1.000 |
| Grade ≥3 immune-related hepatitis | 0 (0.0%) | 0 (0.0%) | 1 (1.9%) | 1 (1.2%) | 0.312 |
| Any-grade chemotherapy-related toxicity | NA | NA | 32 (60.4%) | 32/53 (60.4%) | NA |
| Grade ≥3 chemotherapy-related toxicity | NA | NA | 8 (15.1%) | 8/53 (15.1%) | NA |
| Grade ≥3 leukopenia | NA | NA | 5 (9.4%) | 5/53 (9.4%) | NA |
| Grade ≥3 neutropenia | NA | NA | 4 (7.5%) | 4/53 (7.5%) | NA |
| Grade ≥3 anemia | NA | NA | 1 (1.9%) | 1/53 (1.9%) | NA |
| Grade ≥3 thrombocytopenia | NA | NA | 1 (1.9%) | 1/53 (1.9%) | NA |
| Grade ≥3 nausea/vomiting | NA | NA | 2 (3.8%) | 2/53 (3.8%) | NA |
| Grade ≥3 fatigue | NA | NA | 1 (1.9%) | 1/53 (1.9%) | NA |
| Grade ≥3 peripheral neuropathy | NA | NA | 0 (0.0%) | 0/53 (0.0%) | NA |
| AE-related treatment interruption/delay | NA | 2 (7.1%) | 8 (15.1%) | 10 (12.3%) | NA |
| AE-related chemotherapy dose reduction | NA | NA | 5 (9.4%) | 5/53 (9.4%) | NA |
| AE-related permanent immunotherapy discontinuation | NA | 1 (3.6%) | 2 (3.8%) | 3 (3.7%) | NA |
| AE-related permanent chemotherapy discontinuation | NA | NA | 3 (5.7%) | 3/53 (5.7%) | NA |
| Treatment-related death | 0 (0.0%) | 0 (0.0%) | 0 (0.0%) | 0 (0.0%) | 1.000 |

Note: Values are presented as n (%) unless otherwise indicated. AE, adverse event; NA, not applicable. Adverse events were graded according to CTCAE version 5.0. For immune-related adverse events, P values compare the observation group with the overall immunotherapy-based adjuvant treatment group using Fisher's exact test or Pearson's chi-square test, as appropriate. irAEs in the observation group referred to delayed or persistent immune-related events after neoadjuvant PD-1 inhibitor exposure that were documented during the postoperative observation period. Chemotherapy-related toxicities, chemotherapy dose reduction, and chemotherapy discontinuation were evaluated only among patients receiving adjuvant immunochemotherapy; therefore, the denominator in the overall adjuvant treatment column is 53 for these rows.


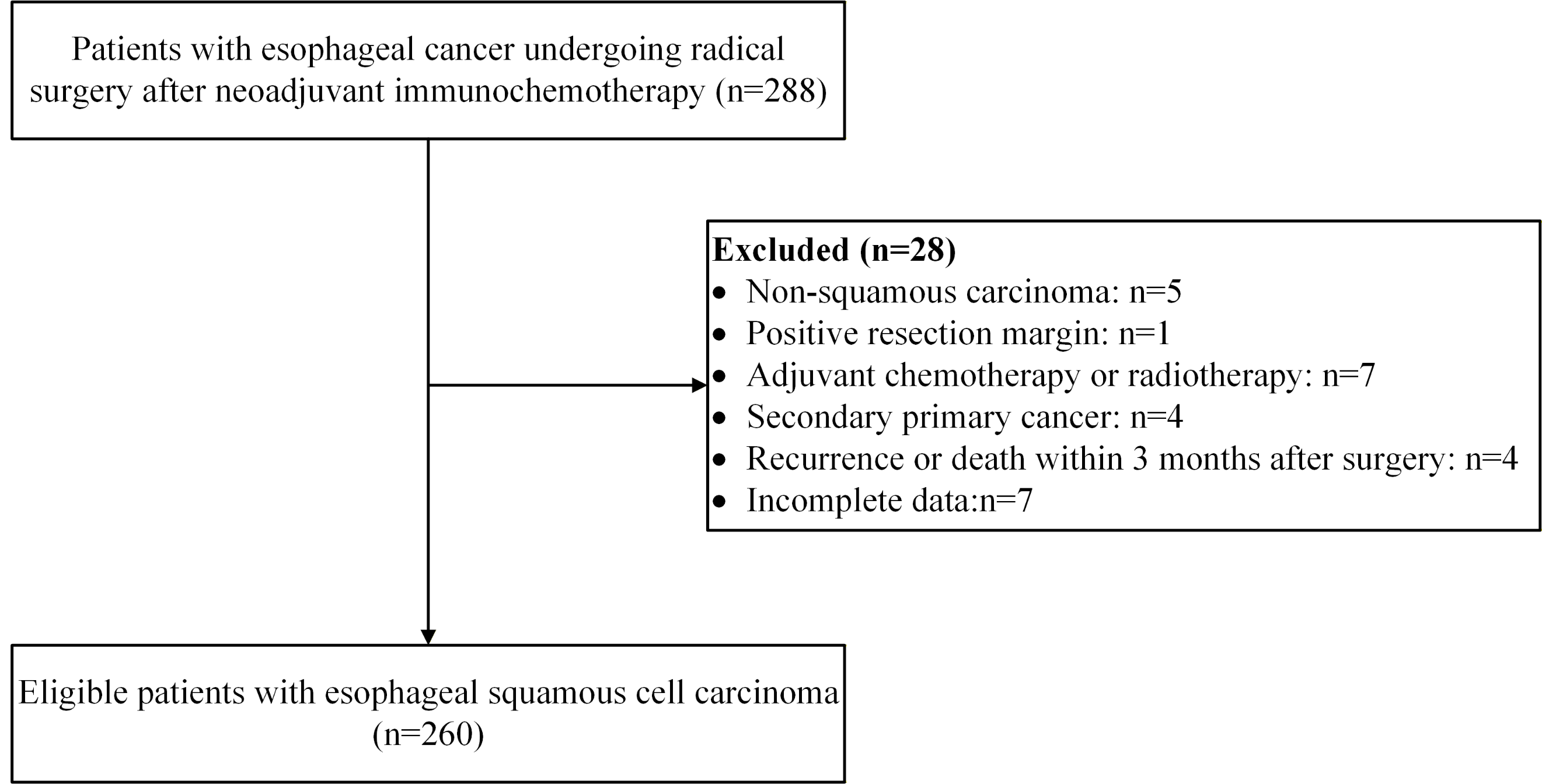


Figure S1. Flow diagram of patient inclusion and exclusion criteria.


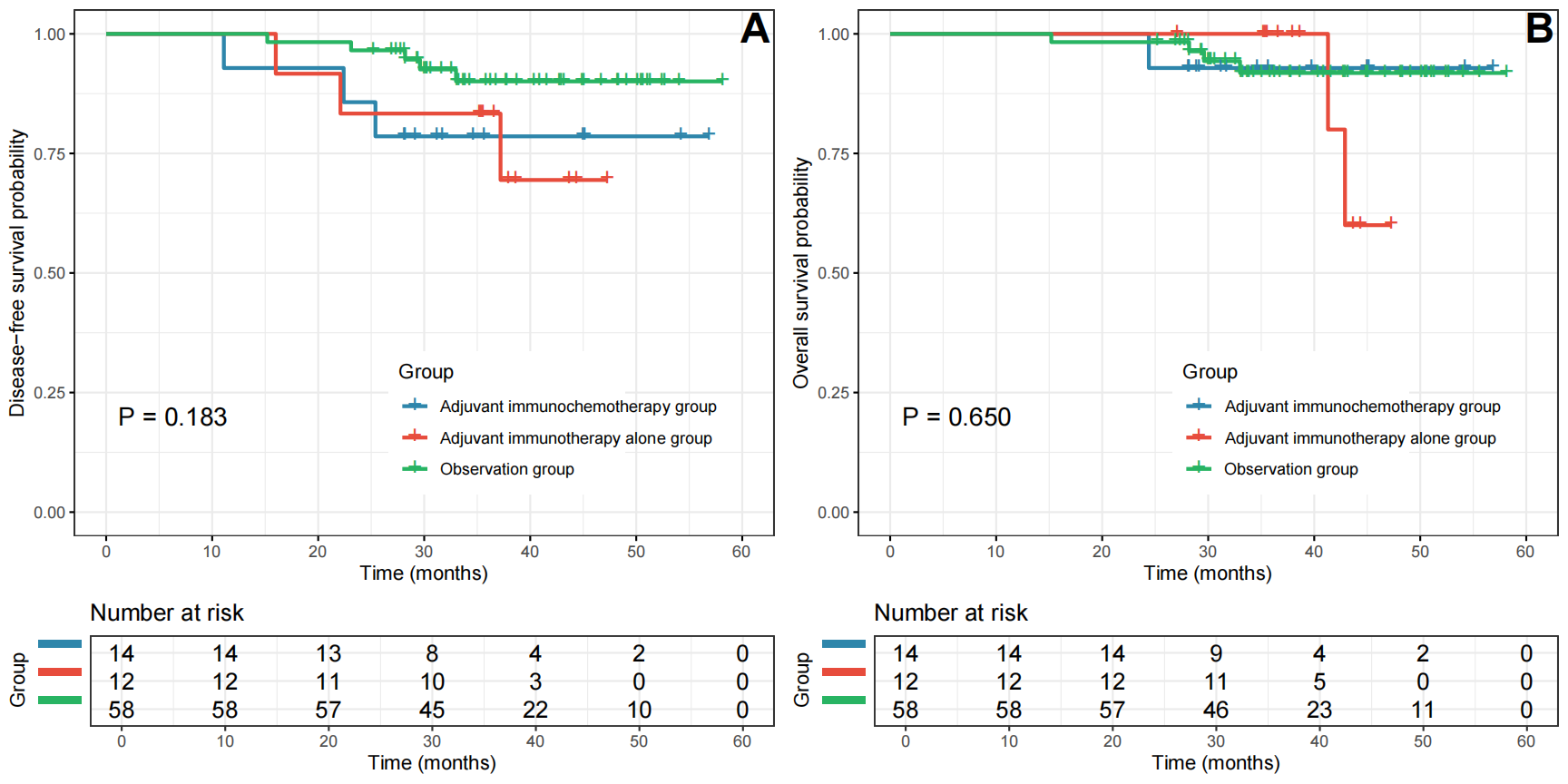


Figure S2. Kaplan–Meier survival curves comparing observation, adjuvant immunotherapy alone, and adjuvant immunochemotherapy groups for disease-free survival (DFS) and overall survival (OS) in the pCR population.


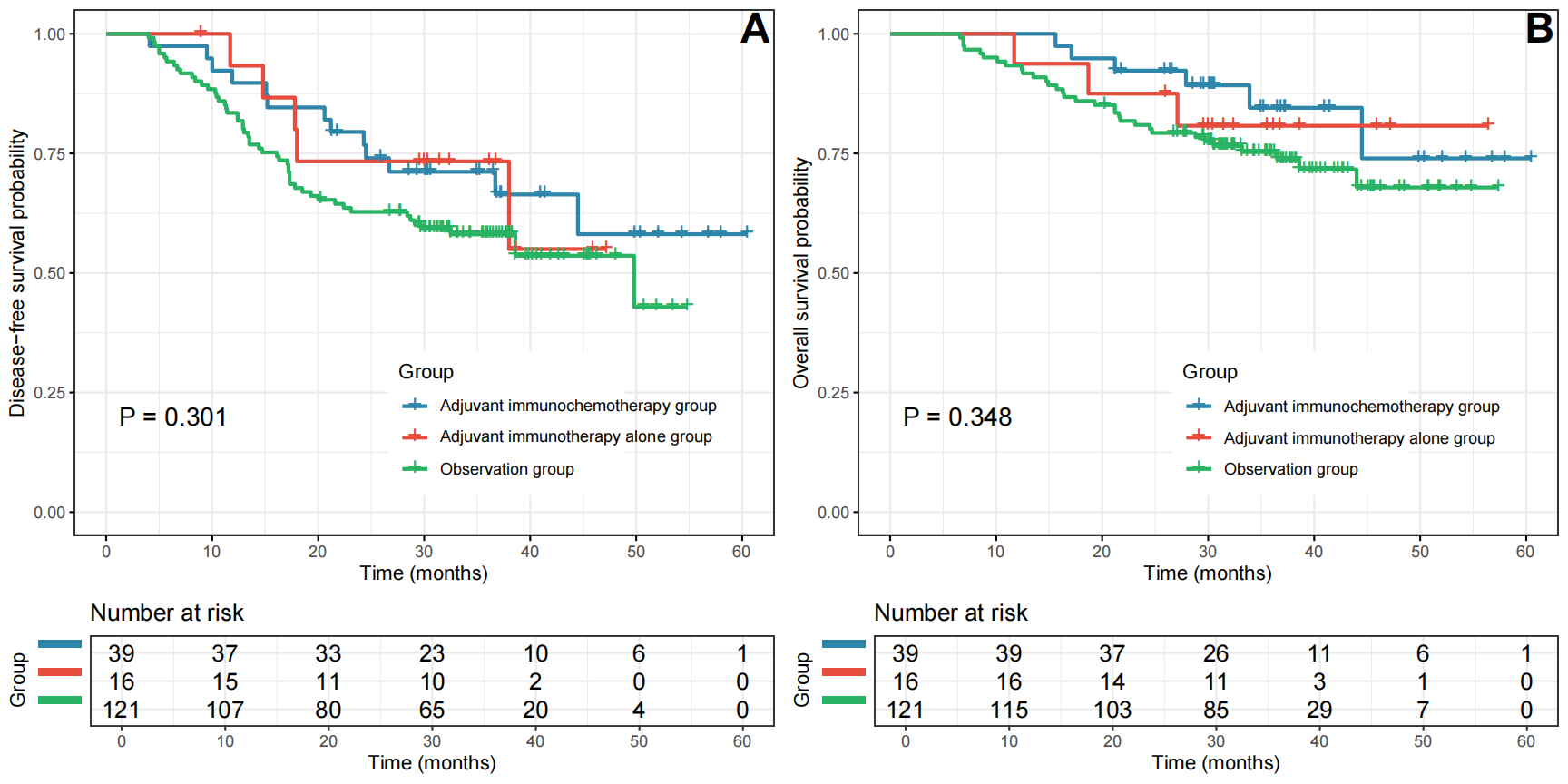


Figure S3. Kaplan–Meier survival curves comparing observation, adjuvant immunotherapy alone, and adjuvant immunochemotherapy groups for disease-free survival (DFS) and overall survival (OS) in the non-pCR population.


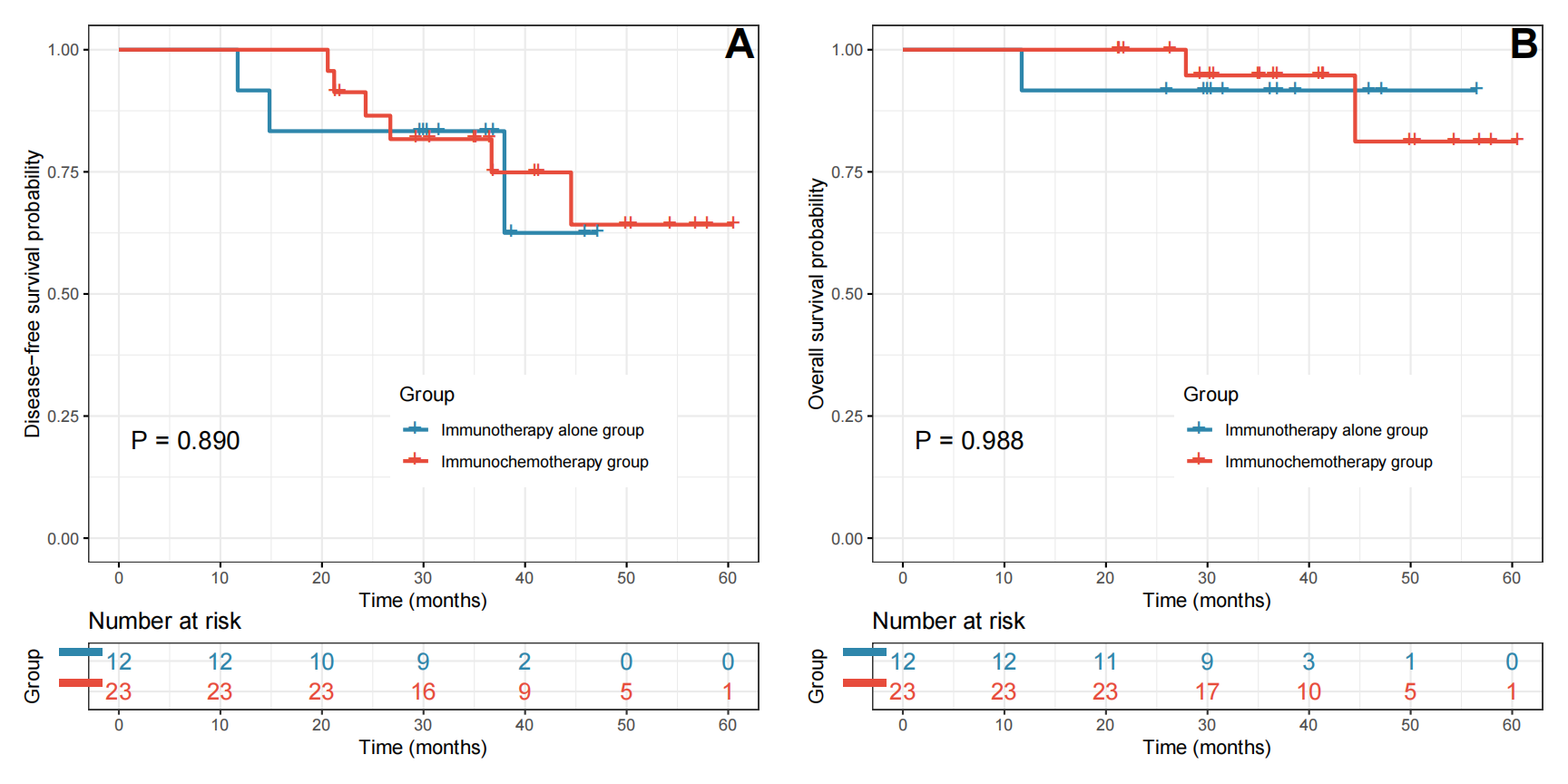


Figure S4. Kaplan–Meier survival curves comparing immunotherapy alone and immunochemotherapy for disease-free survival (DFS) and overall survival (OS) among patients with pathological downstaging in the non-pCR population.
